# Fecal Nitrogen Concentration as a Nutritional Quality Indicator for European Rabbit Ecological Studies

**DOI:** 10.1371/journal.pone.0125190

**Published:** 2015-04-20

**Authors:** Esperanza Gil-Jiménez, Miriam Villamuelas, Emmanuel Serrano, Miguel Delibes, Néstor Fernández

**Affiliations:** 1 Department of Conservation Biology, Estación Biológica, Spanish Council for Scientific Research CSIC, Seville, Spain; 2 Servei d’ Ecopatologia de Fauna Salvatge, Departamento de Medicina y Cirugia Animal, Universidad Autónoma de Barcelona (UAB), Bellaterra, Barcelona, Spain; 3 CESAM, Departamento de Biologia, Universidade de Aveiro, Aveiro, Portugal; Tokai University, JAPAN

## Abstract

Measuring the quality of the nutritional resources available to wild herbivores is critical to understanding trophic regulation processes. However, the direct assessment of dietary nutritional characteristics is usually difficult, which hampers monitoring nutritional constraints in natural populations. The feeding ecology of ruminant herbivores has been often assessed by analyzing fecal nitrogen (FN) concentrations, although this method has been less evaluated in other taxa. This study analyzed the suitability of FN as an indicator of ingesta quality in the European rabbit (*Oryctolagus cuniculus*), which is a keystone lagomorph species in Mediterranean ecosystems and of great conservation interest. Firstly, domestic *O*. *cuniculus* were used to evaluate under experimental conditions the accuracy of total FN and the metabolic FN as diet quality indicators of forages with characteristics similar to those available under natural conditions. Secondly, the accuracy of Near-Infrared Spectroscopy (NIRS) to calculate FN was tested using partial least squares regression. Thirdly, a pilot field study was conducted to monitor FN dynamics from wild *O*. *cuniculus* in three different habitats during wet and drought periods. A strong association was found between diet type and total FN and metabolic FN (*Pseudo-R^2^* ≥ 0.89). It was also found that NIRS calibrations were accurate for depicting nitrogen concentrations (*R^2^* > 0.98 between NIRS and chemical results). Finally, the seasonal FN dynamics measured in the field were consistent with current knowledge on vegetation dynamics and forage limitations in the three habitats. The results support the use of NIRS methods and FN indices as a reliable and affordable approach to monitoring the nutritional quality of rabbit habitats. Potential applications include the assessment of the mechanistic relationships between resource limitations and population abundance, e.g., in relation to natural drought cycles and to habitat interventions aimed at reinforcing rabbit populations.

## Introduction

Diet quality has been identified as a key regulator of the nutritional status, body condition, survival rate, reproductive success, and, ultimately, population dynamics of mammalian herbivore populations (e.g. [1]). Thus, numerous studies have demonstrated the importance of spatial and temporal changes in the amount and quality of available forage on the population abundance of these populations (e.g. [2–5]). It has been suggested that forage quality plays an especially critical role in the nutritional regulation of small-herbivore populations, whereas large-herbivore populations tend to be more constrained by forage quantity. In fact, whereas large herbivores can extract more energy from worse-quality forage, small herbivores have a limited capacity to compensate for reductions in food quality by increasing their intake due to their high energy expenditure per unit mass and short ingesta retention times [6]. Therefore, temporal and spatial variability in the digestive quality of ingested food has to be quantified to understand bottom-up regulation processes in small-herbivore populations and for their management and conservation.

Diet quality in herbivores is often characterized by protein content since the assimilation of nitrogen, a key element of amino acids, determines animal growth [7]. Protein availability may even have a greater limiting role than other food components, such as fats, especially during critical periods of the animal life cycle, such as the pregnancy, lactating, fetal development, and neonatal growth periods [1]. Methods for assessing diet quality based on protein content can be classified into three broad approaches. The first approach is to analyze protein concentrations in vegetation samples directly collected in the field, under the assumption that these samples represent the vegetation available for consumption [8–10]. However, this assumption may not hold if the sampling scheme fails to account for the selective feeding behavior of animals. The second approach consists in analyzing stomach contents, thus ensuring that the analyzed material was actually ingested by the individuals [11–13]. However, this is an invasive technique that requires animals to be dead or killed and is therefore an unfeasible approach in many species because of ethical and conservation concerns. The third approach is to analyze nitrogen content in herbivore feces, which are easy to collect non-invasively under normal field conditions (see [14] for a review).

The use of fecal indicators of diet quality has proven to be a reliable and cost-effective method in ecological studies of ruminants (e.g. bighorn sheep (*Ovis Canadensis*) [15], red and roe deer (*Cervus elaphus* and *Capreolus capreolus*) [16], fallow deer (*Dama dama*) [17], white-tailed deer (*Odocoileus virginianus*) [18], kudu (*Tragelaphus strepciseros*) [19], and roe deer (*Capreolus capreolus*) [20]). These species have a four-chamber stomach that allows the maximum digestion of fibers and the assimilation of nutrients, particularly nitrogen, from forage [14]. Although these indicators have also been used in hindgut fermenters, such as the domestic horse [21] and the African bush elephant (*Loxodonta Africana*) [22], and even in a marsupial folivore, the common brushtail possum (*Trichosurus vulpecula*) [23], the reliability of this approach to the ecological study of the diet of herbivore species with different digestive mechanisms remains relatively unknown.

The analysis of fecal nitrogen concentrations for diet quality assessment in wildlife has been greatly improved by the application of spectroscopic methods. Apart from traditional techniques based on the combustion of samples, such as the Kjeldahl and the Dumas methods [24–26], near-infrared spectroscopy (NIRS) has recently emerged as a highly accurate method to measure chemical compounds in feces ([27], for a review). An important advantage of this method is that, compared to previous methods, it considerably reduces costs and analysis times, which ensures more comprehensive samplings [28,29]. Briefly, NIRS consists in analyzing the spectral reflectance signature in the infrared spectra (wavelengths between 700–2500 nm) produced by the interaction between radiated energy and the molecular bonds of chemical compounds. This signature can then be compared to reference samples of known content, and thus the concentrations of different chemical compounds can be measured [30,31]. NIRS in animal ecology studies has mainly been applied to ruminants [18,27,32,33] and has proven to be a highly cost-efficient technique for analyzing large amounts of field samples.

The present study evaluated the suitability of FN concentrations, and specifically NIRS, to analyze the diet quality of the European rabbit (*Oryctolagus cuniculus*) in ecological studies. The European rabbit is a key prey in Mediterranean ecosystems and an ecosystem engineer of great conservation importance [34,35]. Understanding the effects of diet quality on the population dynamics of the European rabbit is considered critical to the management of the species and its predators [4,8,36–38]. However, this issue is challenging due to the difficulty of disentangling the interactions between nutritional resource availability, predation, and diseases in determining how rabbit populations are regulated [4,8,39]. Specifically, there is a remarkable lack of knowledge on rabbit nutrition in relation to environmental variability, and thus reliable and cost-effective methods are needed to monitor diet quality under natural conditions.

Unlike ruminants, rabbits are monogastric herbivores with a colonic separation mechanism [40]. In addition, they practice caecotrophy, producing two types of feces: soft feces or “caecotrophs”, which are reingested to maximize nutrient assimilation, and hard fecal pellets, which are the final excretion [41–43]. Previous studies that have used NIRS to investigate diet quality in the European rabbit have evaluated the digestibility, chemical composition, and energy values of optimized commercial compound feeds for domestic animals [44–47], whereas no study has yet attempted to analyze whether these powerful techniques are suitable for ecological studies. For this reason, an experimental evaluation is needed of the relationship between protein intake and fecal nitrogen that takes into the account the greater variability in diet quality of rabbits under natural conditions.

We tested the hypothesis that nitrogen concentrations in European rabbit feces is determined by the protein content in the diet. Having confirmed the hypothesis, we attempted to optimize the analytical procedure to obtain an affordable method to analyze large samples in ecological applications. The study was organized in three parts. Firstly, under controlled experimental conditions, we investigated the suitability of fecal biochemical analyses as a means to estimate diet quality in domestic rabbits fed with natural forage similar to that available in their habitats. Secondly, species-specific NIRS calibrations were determined in order to measure FN concentrations in rabbits feeding on highly contrasting forages in terms of protein concentration. Thirdly, the usability of FN concentrations in ecological studies was tested by conducting a pilot study that evaluated the temporal variability in the diet quality of free-living European rabbits in the field.

## Materials and Methods

### Experimental evaluation of fecal nitrogen indicators

Domestic rabbits were used in a controlled feeding trial to test the relationship between FN concentrations and the nutritional quality of ingested food. Domestic rabbits were used instead of wild rabbits because the latter typically show high stress levels during confinement, which may affect both their wellbeing and the results of the experiment. The domestic rabbit originated from the subspecies *O*. *cuniculus cuniculus* about 1500 years ago [48]. Given the close relatedness between both forms, it was assumed that the physiological response to potential changes in diet quality would be similar in wild and domestic rabbits.

Four different forages were tested comprising three herbaceous graminoid species and a legume species that differed in quality according to their protein and fiber contents (Table 1). Diets based on entire plants instead of compound feeds were selected in order to cover the wide range of nutrient concentrations and to simulate the characteristics of forage typically available to wild rabbits in Mediterranean environments.

**Table 1:** Crude protein content (CP) and Neutral Detergent Fiber content (NDF) of feeds used in the experiment.

Feed	Treatment Quality	CP (%)*	NDF (%)
Alfalfa (*Medicago sativa*)	High	18.96	33.57
Tall fescue (*Festuca arundinacea*)	Medium	13.05	61.10
Oat (*Avena sativa*)	Low	9.81	53.93
Mixture of barley (*Hordeum vulgare*) and Oat Straw	Very low	4.86	75.52

* CP was estimated as nitrogen content x 6.25 according to [49].

Twenty individuals were used in the experiment, each being tested with three different treatments (N = 60). Individuals were randomly distributed into four blocks of five animals each. The treatments consisted in habituating the animals to the new diet over 3–5 days followed by an experimental trial of 5 days. Treatment duration was based on previous digestibility studies that showed that 98% clearance is reached within 36±10 h [50]. During the trial, a fixed amount of food was provided 1–2 times per day and non-consumed leftovers and feces were collected at least twice, once in the middle of the trial period and once at the end. The sequence of the three consecutive treatments was the same for all individuals in the same block but differed between blocks.

Rabbits were housed on a 12-h light/12-h dark schedule in individual cages (65x40x36cm) at 20°C. Food and fresh water were offered *ad libitum*. Caecotrophy was not prevented in order to permit fully functional digestion. Prior to the experiment, all rabbits underwent an acclimatization period of 8–10 days during which the diet was gradually changed to the same experimental feed (Alfalfa) to homogenize the starting conditions. During this period, it was ensured that the rabbits did not lose weight and remained healthy.

Forage samples (n = 5 per diet type) were dried to a constant weight to estimate the proportion of dry matter/total weight in each feed. At the end of each trial, the rabbits were moved to a new clean cage and the hard fecal pellets produced during the next hour were collected to analyze the nitrogen content. This method ensured that all the samples represented the final digestion product at the end of the treatment period, thereby minimizing the effects of previous diets on fecal content and avoiding sample contamination (e.g. by urine). Only hard fecal pellets, but not caecotrophes, were collected.

All procedures were conducted in strict compliance with all the applicable Spanish and European animal welfare regulations according to the Spanish Real Decreto 1201/2005 and were also approved by the Animal Health Authority of the Andalusian Government and by the Ethics Committee for Animal Welfare of the Estación Biológica de Doñana, CSIC (#CEBA-EBD_13_62). The experiment was conducted at the animal facilities of the Estación Biológica de Doñana (Animal Experimentation Installation REGA #410910008014). The experiment was designed in such a way to reduce the number of animals used and the 3Rs were implemented to maximize animal welfare. This approach included using domestic animals to minimize stress, optimizing the statistical design to reduce the number of animals and treatment days, providing the animals with optimal caging conditions, and reducing their manipulation.

### Chemical analysis of nitrogen content

Forage and fecal samples were thawed and oven-dried at 60°C for 24 h to a constant weight and subsequently ground with a 1-mm pitch laboratory mill (Cyclotec 1093; FOSS Tecator, Höganäs, Sweden). Each sample was analyzed to determine the concentrations of three constituents: fecal nitrogen (FN), neutral detergent fiber (NDF), and fecal nitrogen bonded to fiber (NDF-N). NDF-N is the nitrogen bound to cell wall components and is considered to be unavailable for digestion. Therefore, the percentage of metabolic fecal nitrogen (MFN) was also calculated as the difference between FN and NDF-N. MFN is assumed to reflect diet digestibility since it includes the amount of nitrogen present in feces due to endogenous losses and symbiotic gut bacteria, which are present at higher concentrations in more digestible diets [51].

Fecal samples were divided into two subsamples to detect possible measurement errors. Matter was dried at 103°, as established by the AOAC [52], and the dry matter weight (DM) and FN, NDF-N, and NDF concentrations were determined for each subsample. FN and NDF-N were determined by the Dumas dry combustion method [25,26] using a LECO analyzer (LECO Corporation, St. Joseph, MI, USA). Fiber concentration (NDF) was determined by the Van Soest technique [53] following the Ankom method [54] (Fiber Analyzer 220, Ankon Technology, Fainport, NY). All concentrations were calculated in relation to DM.

### NIRS analysis of fecal samples

The results of the Dumas method were used to perform the NIRS calibration for each index (i.e. FN, NDF-N, and NDF). Briefly, NIRS calibration compares the chemical signature obtained from the spectroscopic analysis of each sample to the chemical concentrations obtained by the Dumas method [30,31]. The 60 samples described above were used in this procedure plus 14 more samples collected in the field in order to account for the full range of FN concentrations observed in this study (see below).

Fecal samples were scanned from 1100 to 2500 nm using a NIRSystems 5000 scanning monochromator (FOSS, Hillerød, Denmark). Reflectance was recorded at 2-nm intervals as *log(1/R)*, where *R* is reflected energy. WinISI III (v. 1.6) software was used to analyze the spectra and develop chemometric models. Prior to calibration, the values of *log*(1/*R*) were corrected for scatter effects using the standard normal variate (SNV) and/or detrend (DT) method [55] and by multiplicative scatter correction (MSC) [56] to reduce the effects of particle size. The calibrations were performed by modified partial least square regression (PLS) [57] using first and second derivatives of the spectra. Cross-validation was applied to optimize calibration models and to detect outliers. For each predicted parameter, spectral models were developed based on the results of the four scatter correction techniques (SNV: Standard normal variate; DT: detrend; SNV+DT: Standard normal variate and detrend; MSC: Multiplicative scatter correction) and two different mathematical treatments routinely used in NIRS calibration according to the protocol established by Shenk and Westerhaus [57,58] (1.4.4.1; 2.4.4.1-derivate number, subtraction gap, smooth, second smooth). Calibration accuracy was improved by calculating the mean of two scans for each sample.

The following statistics were calculated to determine the capacity of the NIRS model to predict nitrogen concentrations: standard error of calibration (SEC), standard error of cross-validation (SECV), coefficient of determination for the calibration (*R* ^*2*^), coefficient of determination for cross-validation (*r* ^*2*^_*cv*_), the ratio of performance to deviation (RPD, defined as the ratio of the standard deviation of the validation samples to SECV), and the range error ratio (RER, defined as the ratio of the range in the reference data from the validation set to the SECV; for a detailed description, see [59]).

### Field tests

The field study was conducted between February and July 2013 in three different areas of the Doñana National Park (DNP), south-western Spain (37°0’N, 6°30’W). The DNP is a strictly protected area with a Mediterranean sub-humid climate and an average annual precipitation of 550 mm. Climate is characterized by strong seasonality: >80% of the rainfall occurs between October and April, whereas the climate is dry and hot between May and September. Vegetation phenology is characterized by a growth period that peaks between the end of winter and the beginning of spring and by a strong hydric stress period in summer [60]. Prior to the study, all the necessary permits were obtained from the National Park Administration.

Three study areas were selected that were representative of different habitats in the DNP: Mediterranean hydrophytic shrubland dominated by Mediterranean *Pistacia lentiscus* bushes interspersed with short scrub comprising *Halimium halimifolium*, *Stauracanthus genistoides*, and *Cistus libanotis*, and characterized by the limited availability of pastures for feeding (CR1); a transition zone between the aforementioned shrubland and non-flooding pastureland considered to be high-quality rabbit habitat (CR2); and a grassland area dominated by short herbaceous vegetation in slightly nitrified sandy soils (CR3). Rabbit behavior and abundance in these three areas has been studied in relation to the vegetation structure and the seasonal availability and protein content of grasses [4,8]. Thus, it was possible to compare the results obtained in the present study of the fecal analysis of diet quality with findings from these studies.

Twenty sampling plots were distributed in each area at a distance of 25 m from each other. Between February and July 2013, fresh pellet samples were collected each month in each habitat in five of these plots. A minimum of 5 hard fecal pellets were collected in each plot, because preliminary analyses showed that this number was sufficient to obtain sufficient fecal material to ensure the accuracy of the NIRS analysis. Samples were preserved at -22°C before the NIRS laboratory analyses were performed.

### Statistical analyses

Linear mixed models (LMM) were used to evaluate whether FN and MFN were significantly associated with diet quality using these concentrations as the response variables and diet quality as a fixed factor. Individual identity was included as a random effect in order to account for potential differences in individual responses. Likelihood ratio tests (LRT) were used to identify significant differences between each fitted model and a nested null model that excluded the treatment effect (i.e. the random term alone was included).

Nitrogen concentrations in field samples were analyzed by month of collection and study area. Data were analyzed using LMMs, with the area and month as fixed factors and the sampling plot as a random factor. LRTs were also used to identify differences between the fitted model and the nested null model with the random term.

The assumptions underlying LMMs were verified. Models were carried out using the *lme* function of the “nlme” package [61] of the R statistical software version 3.1.1 [62]. In addition, *Pseudo-R* ^*2*^ values were calculated for all significant LMMs as an overall measure of goodness of fit following the procedure described by Nakagawa and Schielzeth [63] and using the *rsquaredGLMM* function of the “MuMln” package [64].

## Results

### Fecal nitrogen concentration as an indicator of nutritional quality

**Fig 1:**
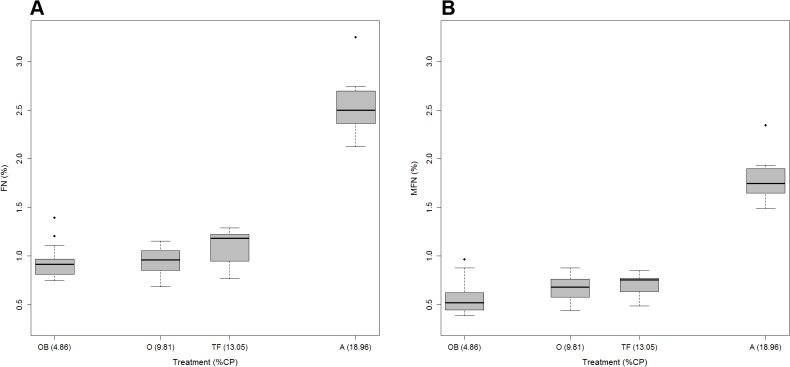
Boxplots of the relationship between nitrogen concentrations and experimental feed. (A) Total fecal nitrogen content by treatment; (B) metabolic fecal nitrogen content. X-axis is proportional to the crude protein content (%) of the treatments. The median is represented by the thick horizontal line; the box is defined by the 25th and 75th percentiles (lower and upper quartile). The dotted line is 1–5 times the spread. Treatments: OB, Oat/barley straw; O, Oat hay; TF, Tall fescue hay; A, Alfalfa hay. *N* = 20 (OB and O); *N* = 10 (TF and A).

Fig 1 shows the relationship found in the experimental trial between diet treatments and FN and MFN concentrations. Likelihood ratio tests confirmed that the treatment had a significant effect on FN (LRT: χ^2^ = 149.47, d.f. = 3, *P*<0.001) and MFN (LRT: χ^2^ = 137.35, d.f. = 3, *P*<0.001). FN and MFN levels increased as dietary protein increased (Table 2 and supplementary material, S1 Table). All LMMs had a high goodness of fit: marginal *pseudo-R* ^*2*^ (i.e. the variability explained by the treatment) was *R* ^*2*^ *m* = 0.91 for FN and *R* ^*2*^ *m* = 0.89 for MFN, thus confirming the strong association between ingested and excreted nitrogen concentrations. In addition, correlation analyses confirmed that FN concentrations are representative of fecal metabolic concentrations (Spearman’s *r* = 0.95 for the correlation between FN and MFN, and *r* = 0.69 for the correlation between FN and NDF-N), therefore confirming the suitability of FN as an indicator of the bioavailable fraction.

**Table 2:** Fixed effects of the linear mixed model for total fecal nitrogen content (FN) and metabolic nitrogen content (MFN) as response variables and treatment as a predictor.

Response variable	Fixed effects	Parameter estimates ± SE	*t-*value
**Total FN**			
	Intercept	0.927 ± 0.042	22.760
	Oat hay	0.015 ± 0.052	0.293
	Tall fescue	0.175 ± 0.065	2.698
	Alfalfa	1.627 ± 0.065	25.020
**Metabolic FN**			
	Intercept	0.558 ± 0.033	16.678
	Oat hay	0.110 ± 0.042	2.592
	Tall fescue	0.160 ± 0.053	3.003
	Alfalfa	1.231 ± 0.053	23.092

Individual identity was included as a random effect. *N* = 60.

### NIRS analyses

NIRS analyses showed that there was a very high correspondence between each of the FN concentration indices and the predictions of the NIRS PLS equations (Table 3; see also supplementary material, S1 Fig). Depending on the index, the most suitable spectral pre-treatments were the first and the second derivate treatment in combination with SNV+DT and MSC. All three calibrations were highly accurate (Table 3). The resulting coefficients of determination of the relationship between nitrogen concentrations estimated by NIRS and the Dumas method were *R* ^*2*^ = 0.993 for FN; *R* ^*2*^: = 0.986 for NDF; and *R* ^*2*^ = 0.980 for NDF-N.

**Table 3:** Calibration and cross-validation statistics of the NIRS results for the estimation of NDF (neutral detergent fiber), FN (fecal nitrogen), and NDF-N (nitrogen bound to fibers) content from wild rabbit (*Oryctolagus cuniculus*) fecal samples.

Calibration set	*N*	Math z^a^ treatment	Scatter ^b^ correction	*R* ^*2*^	SEC	*r* ^*2*^ *CV*	SECV	RPD	RER
NDF	74	1,4,4,1	SNV+DT	0.986	1.064	0.970	1.472	5.715	31.071
FN	74	1,4,4,1	MSC	0.993	0.050	0.986	0.069	8.632	37.145
NDF-N	73	2,4,4,1	SNV+DT	0.980	0.026	0.950	0.039	4.390	17.256

^a^ Values refer to: derivative order, gap (number of data points over which derivation was computed), first smoothing (number of data points for spectral smoothing), and second smoothing (data points for second spectral smoothing), respectively.

^b^ Standard Normal Variate (SNV), Detrend (DT), Multiple Scatter Correction (MSC) transformations

Abbreviations: *N*, Number of spectra; *R* ^*2*^, coefficient of determination for calibration; SEC, standard error of calibration; SECV, standard error of cross validation; SD, standard deviation; *r* ^*2*^ *CV*, coefficient of determination for cross validation; RPD, ratio SD of calibration data set to SECV; RER, ratio range (maximum-minimum) of calibration data set to SECV.

### Field study

Given the above-mentioned results, field tests were restricted to NIRS FN as an overall indicator of the nutritional quality of ingested food. The variability in FN concentrations measured in the field (0.71–3.59%) was very similar to the range of values obtained in the laboratory using controlled feeds (0.69–3.25%). However, 6 samples (i.e. 7.2% of field samples) had higher concentrations than those observed in the experiment.

**Fig 2:**
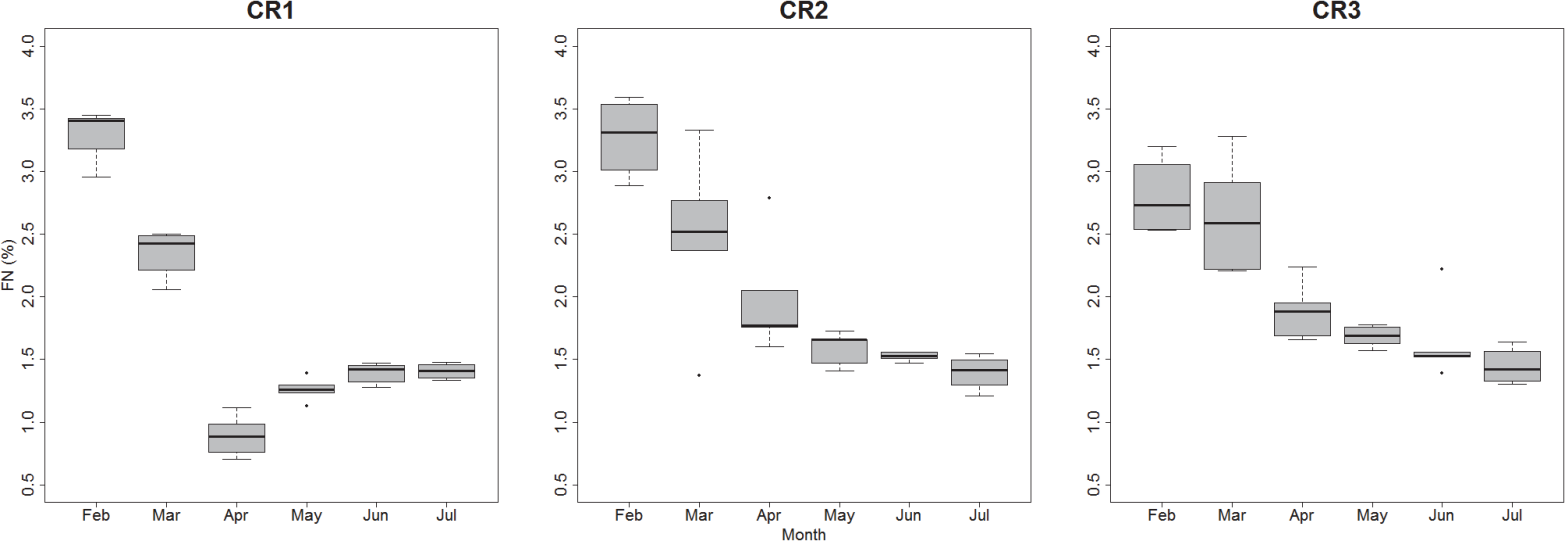
Boxplots of total fecal nitrogen (%) from the 3 study areas (February to July 2013). CR = Coto del Rey. The median is represented by the thick horizontal line; the box is defined by the 25th and 75th percentiles (lower and upper quartile). The dotted line is 1–5 times the spread.

A strongly significant relationship was found between field FN concentrations and the study area and month (LRT: χ^2^ = 121.17, d.f. = 7, *P*<0.0001; *R* ^*2*^ *m* = 0.85). As expected, the data showed that FN consistently underwent an overall decrease from February to July in all study areas (Fig 2; Table 4). Maximum values were reached in February in CR1 (%FN = 3.45) and CR2 (%FN = 3.59) and in March in CR3 (%FN = 3.28). Minimum values were observed in July in CR2 (%FN = 1.21) and CR3 (%FN = 1.30) and in April in CR1 (%FN = 0.710). Negligible differences were found between CR2 and CR3 in the temporal course of FN concentrations, although these two areas significantly differed from CR1 (Table 4).

**Table 4:** Fixed effects of the linear mixed model for total fecal nitrogen as the response variable and month and area as the predictor.

Fixed effects	Parameter estimates ± SE	*t* value
Intercept	2.873 **±** 0.122	23.474
March	-0.598 **±** 0.142	-4.212
April	-1.492 **±** 0.139	-10.665
May	-1.569 **±** 0.139	-11.216
Jun	-1.561 ± 0.139	-11.161
Jul	-1.664 ± 0.147	-11.32
CR2	0.317 ± 0.096	3.307
CR3	0.306 ± 0.096	3.186

The individual sampling plot was included as a random effect

## Discussion

The main purpose of this study was to evaluate if FN concentrations are suitable predictors of diet protein content in the European rabbit, which is a lagomorph species of key ecological and conservation importance. A distinguishing aspect of the study was the analysis of this relationship under controlled experimental conditions that took into account the wide range of protein concentrations in the species’ natural diet. Firstly, the high correlation found between fecal nitrogen and MFN confirmed that total FN mainly reflected the nitrogen fraction resulting from endogenous and bacterial activity that represents digestibility. Furthermore, it was found that the total fecal nitrogen was more strongly correlated with the percentage of metabolic fecal nitrogen than with the percentage of nitrogen bound to plant cell wall, which is unavailable for digestion. This finding meets the underlying assumption that FN can be used as an indicator of habitat quality and digestive efficiency in herbivores [51]. Secondly, a strong positive association was found between FN and the quality of the forage supplied; there was a 74.37% decrease in FN between the richest-protein treatment (alfalfa) and the poorest treatment (barley and oat straw). Owing to the characteristics of the digestive system of the European rabbit, it was necessary to assess this relationship in order to use FN concentrations as a nutritional proxy for monitoring the nutritional status of this species.

NIRS calibrations performed under controlled feed conditions resulted in highly accurate predictions for the three estimated parameters: FN, NDF and NDF-N. Regarding the coefficients of determination, the values obtained were similar to or even greater than those reported for crude protein in rabbits receiving compound feeds, i.e. with much lower variability in the protein content [47]. The SECV values for FN were very low (SECV = 0.069), which indicated that the prediction obtained by the NIRS model was highly accurate. This value was even lower than SECV values found in wild ungulates, such as red deer and roe deer (0.147 and 0.12, respectively; [16]), and also lower than the SECV value found in domestic rabbits using protein-rich compound feeds (SECV = 0.22 in crude protein [47]). All the calibration diagnostic values were higher than the minimum values usually considered suitable for prediction (i.e., RER > 10; RPD > 3; and *r* ^*2*^ *cv* > 0.9; [58,59,65]).

In addition to conducting laboratory tests, it was also evaluated whether the diet quality of field rabbits studied in three different areas was consistent with predictions based on current knowledge of vegetation dynamics and, in particular, the availability and quality of forage usually consumed by rabbits [8]. It is worth mentioning that fecal concentrations obtained from field samples during the transition period from wet to drought conditions were generally within the range of concentrations obtained in the domestic rabbit experiment, thus supporting the translation of experimental results to field analyses. Specifically, it was predicted that higher fecal nitrogen concentrations would increase between winter and spring following the typical vegetation productivity cycle in the Doñana National Park [60]. Furthermore, it was predicted that there would be higher fecal nitrogen concentrations in feces obtained in ecotone and grassland habitats according to previous studies based on vegetation analyses, which found that there was a higher availability of protein-rich pastures in these habitats [8,39]. We detected an overall decrease in FN following the predicted seasonal changeover between the end of winter and the beginning of summer. This decrease was generally consistent in all the study areas and particularly consistent in ecotone and grassland (CR2 and CR3, respectively), in which a gradual exponential decay in FN was observed. There were similar decreases in FN in feces obtained in the Mediterranean hydrophytic shrubland (CR1) between February and July. Mediterranean shrubland is characterized by a lower pasture availability and by lower quality according to protein concentrations [8], which was also reflected by the FN levels observed in this study. However, a particularly dramatic decrease in FN was observed in April in this area followed by a slight increase, whereas FN levels obtained during summer were similar to those found in the other two study areas (Fig 2). The magnitude of the decrease in April was unexpected and requires a more detailed analysis.

Although some authors have expressed concern regarding the use of FN as diet quality indicator due to the potential effects of secondary compounds such as tannins [66,67], these effects have been mainly described in browsing species and not in grazers like the European rabbit [20]. In addition, it has been claimed that the effect of tannins can be disregarded when analyzing temporal changes in diet quality in the same population or when analyzing differences between populations living in similar habitats [68].

In conclusion, the laboratory tests showed that FN as measured by NIRS is a useful and reliable index for measuring protein intake by rabbits under very different dietary conditions. This finding was also confirmed by field tests in which food intake was unknown but where the dynamics of FN in three different study areas were consistent with current knowledge on differences in the availability and quality of food. The results open new approaches to the non-invasive study of rabbit nutrition in the field, which is a topic that remains little studied despite its great relevance to understanding rabbit population trends. Monitoring rabbit nutrition from feces would be extremely useful for studying the efficiency of measures aimed at recovering rabbit populations such as managing vegetation to increase the habitat quality or the use of supplemental feeding [69]. Furthermore, the assessment of diet quality by fecal monitoring would lead to a better understanding of the trophic limitation of rabbit populations, e.g. in relation to drought, which is a factor that may become more important given that the severity and frequency of drought are expected to increase in Mediterranean environments [70].

## Supporting Information

S1 Fig Correlations between the measured values and predicted values of the three fecal indices; A) FN; B) NDF; C) NDF-N. (TIF)

S1 Table Mean values and standard deviations of total fecal nitrogen (FN), nitrogen bound to neutral detergent fiber (NDF-N), and metabolic fecal nitrogen (MFN). *N* represents the number of individual rabbits used in each treatment.
(DOC)
