# Supplementary figures and images for: Fecal Nitrogen Concentration as a Nutritional Quality Indicator for European Rabbit Ecological Studies
Source: PLoS One. 2015 Apr 20;10(4):e0125190. doi: 10.1371/journal.pone.0125190 (PMC4404320; doi:10.1371/journal.pone.0125190)

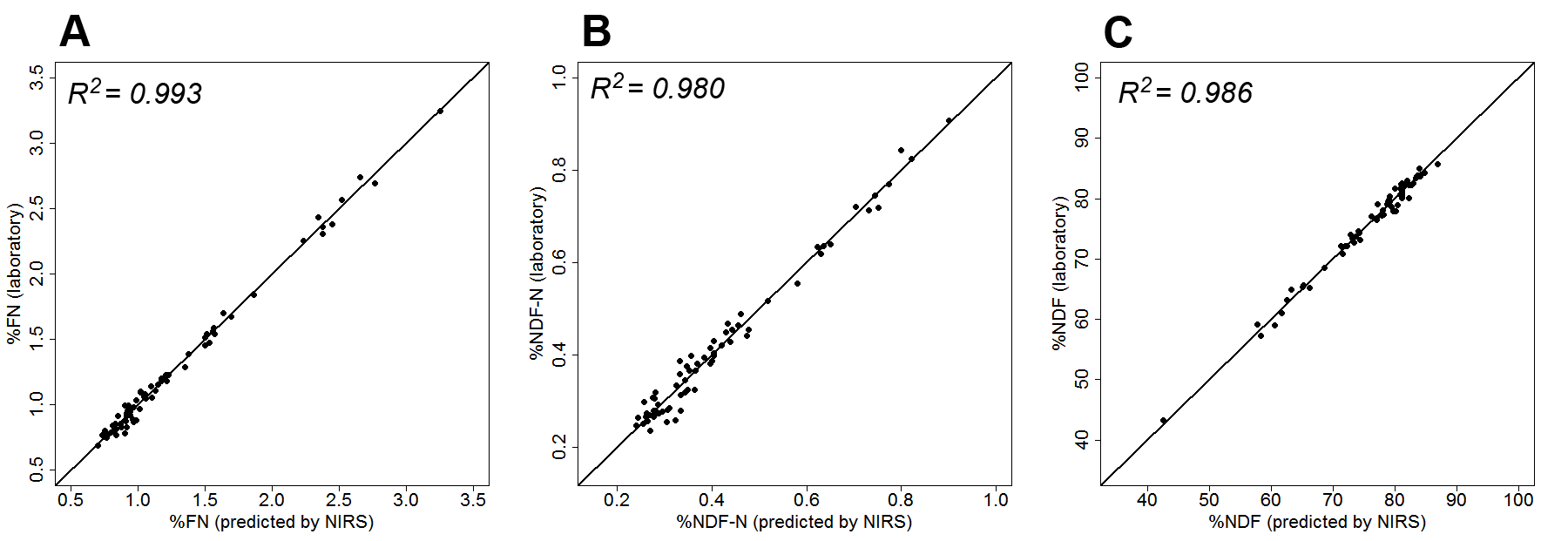

Supplement: S1 Fig — (TIF) [file pone.0125190.s001.tif]
