# Supplementary material for: Fecal Nitrogen Concentration as a Nutritional Quality Indicator for European Rabbit Ecological Studies
Source: PLoS One. 2015 Apr 20;10(4):e0125190. doi: 10.1371/journal.pone.0125190 (PMC4404320; doi:10.1371/journal.pone.0125190)
Supplement: S1 Table — N represents the number of individual rabbits used in each treatment. (DOC) [file pone.0125190.s002.doc]

**S1 Table. Mean values and standard deviations of total fecal nitrogen (FN), nitrogen bound to neutral detergent fiber (NDF-N) and metabolic fecal nitrogen (MFN).** *N* represents the number of individual rabbits used in each treatment.

| Diet | *N* | FN (%DM) | NDF-N (%DM) | MFN (%DM) |
| --- | --- | --- | --- | --- |
| Oat/barley straw | 20 | 0.93 ± 0.16 | 0.37 ± 0.05 | 0.56 ± 0.15 |
| Oat | 20 | 0.94 ± 0.13 | 0.27 ± 0.02 | 0.67 ± 0.12 |
| Tall fescue | 10 | 1.10 ± 0.18 | 0.39 ± 0.08 | 0.71 ± 0.11 |
| Alfalfa | 10 | 2.56 ± 0.31 | 0.76 ± 0.09 | 1.79 ± 0.24 |
